# Impact of baseline SARS-CoV-2 antibody status on syndromic surveillance and the risk of subsequent COVID-19—a prospective multicenter cohort study

**DOI:** 10.1186/s12916-021-02144-9

**Published:** 2021-10-14

**Authors:** Philipp Kohler, Sabine Güsewell, Marco Seneghini, Thomas Egger, Onicio Leal, Angela Brucher, Eva Lemmenmeier, J. Carsten Möller, Philip Rieder, Markus Ruetti, Reto Stocker, Danielle Vuichard-Gysin, Benedikt Wiggli, Ulrike Besold, Stefan P. Kuster, Allison McGeer, Lorenz Risch, Andrée Friedl, Matthias Schlegel, Pietro Vernazza, Christian R. Kahlert

**Affiliations:** 1 Cantonal Hospital St. Gallen, Division of Infectious Diseases and Hospital Epidemiology, Rorschacherstrasse 95, 9007 St. Gallen, Switzerland; 2 Cantonal Hospital St. Gallen, Clinical Trials Unit, St. Gallen, Switzerland; 3 Epitrack, Recife, Brazil; 4 Department of Economics, University of Zurich, Zurich, Switzerland; 5 Psychiatry Services of the Canton of St. Gallen (South), St. Gallen, Switzerland; 6 Clienia Littenheid AG, Private Clinic for Psychiatry and Psychotherapy, Littenheid, Switzerland; 7 Center for Neurological Rehabilitation, Zihlschlacht, Switzerland; 8 Hirslanden Clinic, Zurich, Switzerland; 9 Fuerstenland Toggenburg Hospital Group, Wil, Switzerland; 10 Thurgau Hospital Group, Division of Infectious Diseases and Hospital Epidemiology, Münsterlingen, Switzerland; 11 Cantonal Hospital Baden, Division of Infectious Diseases and Hospital Epidemiology, Baden, Switzerland; 12 Geriatric Clinic St. Gallen, St. Gallen, Switzerland; 13 Federal Office of Public Health, Bern, Switzerland; 14 Sinai Health System, Toronto, Canada; 15 Labormedizinisches Zentrum Dr Risch, Schaan, Liechtenstein; 16 Clienia Littenheid AG, Private Clinic for Psychiatry and Psychotherapy, Littenheid, Switzerland; 17 Center of Laboratory Medicine, University Institute of Clinical Chemistry, University of Bern, Inselspital, Bern, Switzerland; 18 Children’s Hospital of Eastern Switzerland, Division of Infectious Diseases and Hospital Epidemiology, St. Gallen, Switzerland

**Keywords:** COVID-19, Surveillance, Re-infection, Healthcare workers

## Abstract

**Background:**

In a prospective healthcare worker (HCW) cohort, we assessed the risk of SARS-CoV-2 infection according to baseline serostatus.

**Methods:**

Baseline serologies were performed among HCW from 23 Swiss healthcare institutions between June and September 2020, before the second COVID-19 wave. Participants answered weekly electronic questionnaires covering information about nasopharyngeal swabs (PCR/rapid antigen tests) and symptoms compatible with coronavirus disease 2019 (COVID-19). Screening of symptomatic staff by nasopharyngeal swabs was routinely performed in participating facilities. We compared numbers of positive nasopharyngeal tests and occurrence of COVID-19 symptoms between HCW with and without anti-nucleocapsid antibodies.

**Results:**

A total of 4812 HCW participated, wherein 144 (3%) were seropositive at baseline. We analyzed 107,807 questionnaires with a median follow-up of 7.9 months. Median number of answered questionnaires was similar (24 vs. 23 per person, *P* = 0.83) between those with and without positive baseline serology. Among 2712 HCW with ≥ 1 SARS-CoV-2 test during follow-up, 3/67 (4.5%) seropositive individuals reported a positive result (one of whom asymptomatic), compared to 547/2645 (20.7%) seronegative participants, 12 of whom asymptomatic (risk ratio [RR] 0.22; 95% confidence interval [CI] 0.07 to 0.66). Seropositive HCWs less frequently reported impaired olfaction/taste (6/144, 4.2% vs. 588/4674, 12.6%, RR 0.33, 95% CI 0.15–0.73), chills (19/144, 13.2% vs. 1040/4674, 22.3%, RR 0.59, 95% CI 0.39–0.90), and limb/muscle pain (28/144, 19.4% vs. 1335/4674, 28.6%, RR 0.68 95% CI 0.49–0.95). Impaired olfaction/taste and limb/muscle pain also discriminated best between positive and negative SARS-CoV-2 results.

**Conclusions:**

Having SARS-CoV-2 anti-nucleocapsid antibodies provides almost 80% protection against SARS-CoV-2 re-infection for a period of at least 8 months.

**Supplementary Information:**

The online version contains supplementary material available at 10.1186/s12916-021-02144-9.

## Background

Effective and durable host immunity directed against severe acute respiratory syndrome (SARS-CoV-2) is key to the long-term control of the current coronavirus disease 2019 (COVID-19) pandemic. In consequence, the degree and duration of protection against re-infection in those with specific antibodies against SARS-CoV-2 are currently being debated [1]. Documented cases of re-infection (mean interval between infections was 106 days) are increasing and alternative avenues for immunity to SARS-CoV-2 have been proposed [2]. However, recent evidence suggests that neutralizing antibodies against SARS-CoV-2 are consistently detectable for at least 9 months and offer protection against clinically relevant re-infection [3–5]. The most compelling evidence comes from a UK study, where—among 12,000 healthcare workers (HCWs) with a follow-up of 6 months—those with detectable anti-spike antibodies at baseline were less likely to have SARS-CoV-2 detected in a subsequent nasopharyngeal swab [4]. However, this study has not specifically assessed the frequency of COVID-19 specific symptoms among participants.

This HCW cohort study prospectively evaluated the risk of SARS-CoV-2 infection and the occurrence of COVID-19 symptoms among participants with and without SARS-CoV-2 anti-nucleocapsid antibodies at baseline.

## Methods

### Study design

We initiated a prospective cohort study in 23 healthcare institutions in Northern and Eastern Switzerland, before emergence of the second COVID-19 wave in the country (Additional file 1: Figure S1).

Any hospital employee aged 16 years or older with or without patient contact was eligible for the study. Baseline results have been previously reported [6].

Upon study inclusion, participants provided blood for baseline serology. Subsequently, participants were tested through nasopharyngeal swabs (NPS) as soon as they experienced any COVID-19 compatible symptoms such as fever and/or the presence of any respiratory symptom (i.e., shortness of breath, cough, or sore throat). This symptom-based screening strategy was routinely implemented outside the study protocol in all participating institutions according to the recommendations of the Federal Office of Public Health. Also, HCWs residing in a bordering region of Austria or Germany were repetitively tested, irrespective of symptoms.

Via web-based questionnaire, participants responded to questions on demographics and occupation at baseline [6]. Participants were then prospectively followed and reminded by email and/or SMS to complete weekly web-based questionnaires. These collected data on COVID-19 compatible symptoms (syndromic surveillance) and date/result of any SARS-CoV-2 nasopharyngeal swab (NPS) performed by polymerase chain reaction (PCR) or rapid antigen test (Additional file 1: Figure S1). Questionnaires submitted within 2 weeks from baseline serology were excluded from the analysis in order to avoid the detection of symptoms or NPS results associated with episodes, which had started before baseline. Participants were included up to the week where they reported having received their first dose of any SARS-CoV-2 vaccine or up to the end of the observation period, whichever came first.

### SARS-CoV-2 diagnostics

Details of serology testing at baseline are described elsewhere [6]. In brief, venous blood samples were analyzed with an electro-chemiluminescence immunoassay (ECLIA, Roche Diagnostics, Rotkreuz, Switzerland, qualitative detection of total antibodies directed against the nucleocapsid-(N)-protein of SARS-CoV-2), a widely used high-quality test with excellent sensitivity (87.7% at 21 days after infection) and specificity (100%) [7]. Participants were informed about their individual serology result.

Detection of SARS-CoV-2 from NPS was made by PCR or rapid antigen test, depending on the method used in the participating institutions. To verify the completeness and accuracy of self-reported NPS results, all self-reported positive tests and a random sample of negative test results were cross-checked with the database of the division of occupational health for a subgroup of HCWs from the largest participating institution.

### Data analysis

For the primary analysis, we compared the proportion of HCWs reporting at least one positive NPS result between the initially seropositive and seronegative individuals. This analysis was performed using (i) HCWs who had at least one NPS done (i.e., NPS group) and (ii) all HCWs irrespective of NPS testing (i.e., full cohort) as denominator (Additional file 1: Figure S1). Risk ratios (RR) and corresponding 95% confidence interval (CI) were calculated. For the full cohort, we also used a Kaplan-Meier curve to plot the time to positive NPS for seropositive and seronegative HCW; log-rank test was used to compare survival curves between groups; Cox regression was used to calculate hazard ratios (HR) and corresponding CIs. Furthermore, we compared the proportion of HCWs reporting at least one NPS result during follow-up and, among these, the mean number of NPS reported per person. Two sample proportion tests or Wilcoxon rank sum tests were used for this analysis.

Because previously seropositive participants might less frequently undergo NPS testing than seronegative HCW, we also compared the frequency of self-reported symptoms according to serostatus at baseline within the full cohort. For each participant, we summarized a symptom as present if reported in any of the submitted questionnaires, and absent otherwise; risk ratios (and 95% CI) and proportion tests were calculated for each symptom.

To assess specificity of symptoms regarding SARS-CoV-2 infection, we compared the frequency of symptoms between episodes with positive and negative NPS results. Only HCWs symptomatic at time of NPS testing were included. Symptoms reported together with the NPS result and those reported in the previous and following questionnaires were linked to the respective episode. If a participant reported several swabs with the same result, only the first positive and/or the first negative swab were considered. Thus, a participant contributed a maximum of one negative and one positive episode. In participants reporting both positive and negative NPS results, only negative swabs preceding the first positive swab by at least 2 weeks were included as a negative episode; any negative swabs following a positive swab were ignored. Odds ratios (OR) and 95% CIs were estimated for symptom specificity along with Fisher’s exact tests. Analyses were performed with R statistical software, version 4.0.2.

## Results

Between 22 June and 20 October 2020, we recruited 4812 HCW from 17 institutions across Northern and Eastern Switzerland, corresponding to 28% of the eligible HCW population (*n* = 17,060). Of the 4812 HCW, 78% were female and median age was 38.9 years; most worked as nurses (47%) or physicians (17%) (Table 1). These figures were similar to the characteristics of the eligible population, where 76% were female, median age was 40 years, and 40% worked as nurses and 15% as physicians [6].

**Table 1:** Distribution of baseline characteristics among the study participants, and distribution of SARS-CoV- 2 serostatus for each level of the factors

	Total ***N***	Seropositive (***N*** and %)	Seronegative (***N*** and %)
	***N*** = 4812	***N*** = 144	***N*** = 4668
Gender			
Female	3759	108 (2.9%)	3651 (97.1%)
Male	1009	36 (3.6%)	973 (96.4%)
Undetermined/missing	44		
Age in years, median (IQR)	38.9 (30.1–49.8)	35.8 (27.4–45.1)	39.0 (30.3–49.8)
BMI (kg m^−2^ kg/m2), median (IQR)	23.4 (21.3–26.1)	24.0 (22.0–26.7)	23.4 (21.2–26.1)
Comorbidity			
No (none mentioned)	3079	86 (2.8%)	2993 (97.2%)
Yes	1715	58 (3.4%)	1657 (96.6%)
Missing	18		
Profession			
Nurse or medical assistant	2279	88 (3.9%)	2191 (96.1%)
Physician	809	13 (1.6%)	796 (98.4%)
Other	1493	31 (2.1%)	1462 (97.9%)
None/missing	231		
Speciality			
Internal medicine	1025	34 (3.3%)	991 (96.7%)
Surgery/orthopedics	466	15 (3.2%)	451 (96.8%)
Intensive care	325	8 (2.5%)	317 (97.5%)
Emergency department	267	9 (3.4%)	258 (96.6%)
Other	611	15 (2.5%)	594 (97.5%)
None/missing	2121		
Patient contact (medical or administration)			
No	771	12 (1.6%)	759 (98.4%)
Yes	3755	119 (3.2%)	3636 (96.8%)
Missing	286		
Involved in AGP			
No	3307	90 (2.7%)	3217 (97.3%)
Yes	1487	54 (3.6%)	1433 (96.4%)
Missing	18		

*Abbreviations*: *IQR* interquartile range, *BMI* body mass index, *AGP* aerosol-generating procedure

At baseline, 144 (3%) participants were seropositive. Participants were followed until 9 March 2021, equaling a median follow-up of 7.9 months (interquartile range [IQR] 6.7–8.2 months). We received a total of 107,807 weekly questionnaires from these 4812 participants, corresponding to a response rate of 0.71 diaries per person and week. The median number of symptom diaries submitted before vaccination was 24 questionnaires (IQR 14–29) for initially seropositive and 23 (IQR 15–29) for seronegative participants (*P* = 0.83).

A total of 5318 NPS were performed during follow-up, including 3391 (64%) were PCR and 1879 (36%) were antigen tests; 2712 individuals reported having at least one NPS performed (i.e., NPS group). Seropositive participants were less likely (67/144, 47%) to undergo NPS testing than seronegative participants (2645/4668, 57%) (*P* = 0.02). Conversely, the mean number of NPS per person (among those with at least one NPS) did not differ significantly between seropositive and seronegative HCWs (1.8 vs. 2.0 tests, *P* = 0.34). Also, the proportion of antigen tests (vs. PCR) was similar between seropositive (39%) and seronegative HCWs (36%) (*P* = 0.69).

**Fig. 1:**
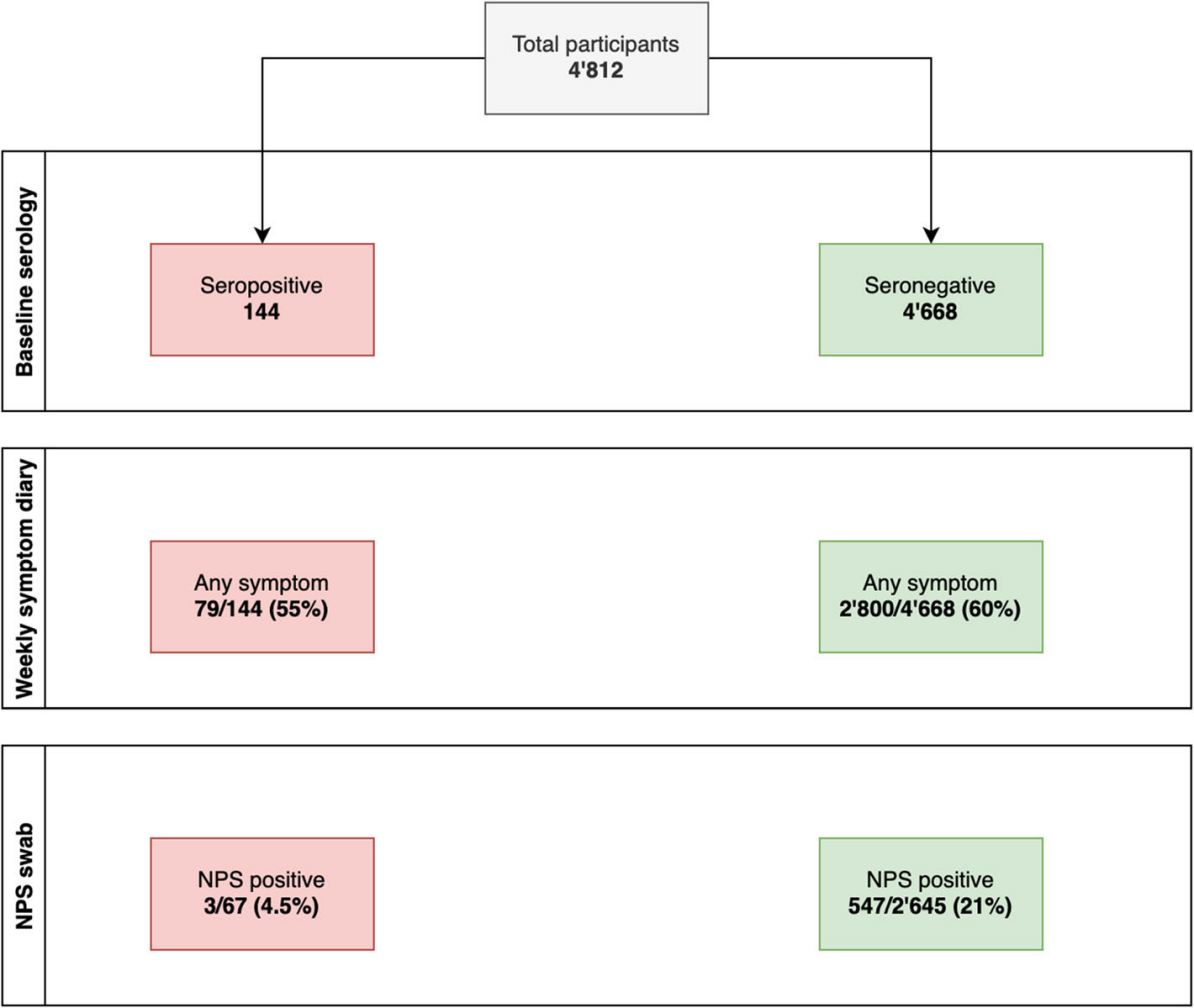
Flow chart, illustrating the distribution of healthcare workers who reported any COVID-19 compatible symptoms and who had a positive nasopharyngeal swab for SARS-CoV-2, according to baseline serology result. Abbreviations: NPS= nasopharyngeal swab. **Colours**: green = baseline seronegative, red = baseline seropositive

**Fig. 2:**
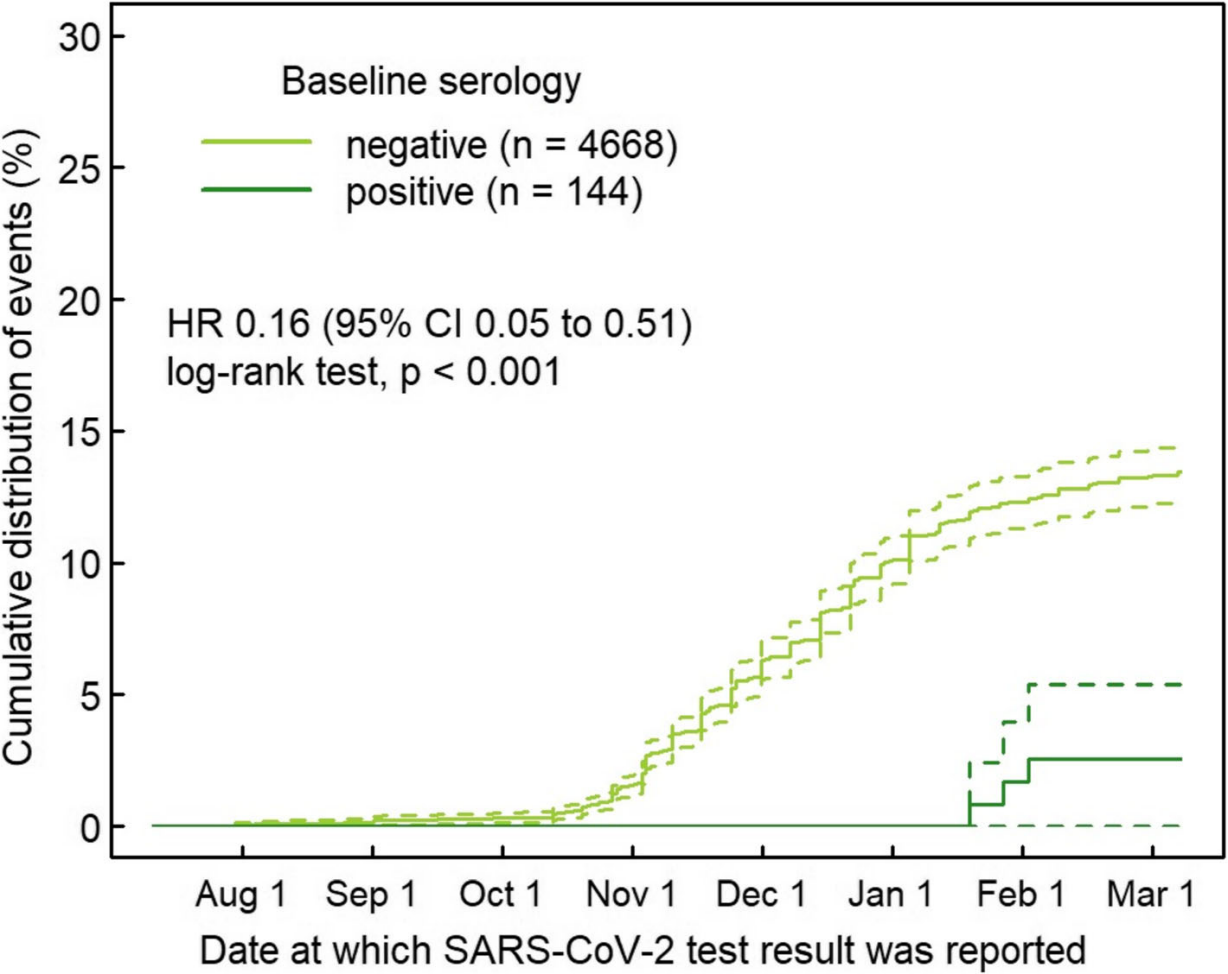
Kaplan-Meier curve showing time to positive nasopharyngeal swab in 4812 healthcare workers with positive (dark green, *n* = 144) and negative (light green, *n* = 4668) baseline anti-nucleocapsid antibodies. Dashed lines show 95% confidence intervals. Abbreviations: HR = hazard ratio, CI = confidence interval, SARS-CoV-2 = Severe Acute Respiratory Syndrome Coronavirus-2

In total, 550 of 2712 participants in the NPS group reported at least one positive NPS during follow-up. Of 67 seropositive participants in the NPS group, only three (4.5%) received at least one positive NPS result during follow-up, compared with 547 (20.7%) of the 2645 seronegative participants (Fig. 1). This translates into a RR of 0.22 (95% CI 0.07 to 0.66, *P* = 0.002) for a positive NPS after positive baseline serology. Including HCW who did not undergo NPS testing (i.e., analysis of full cohort), the corresponding RR was 0.18 (95% CI 0.06 to 0.55, *P* < 0.001). In Cox regression, this translated into a hazard rate of 0.16 (95% CI 0.05 to 0.51, *P* < 0.001) for the full cohort (Fig. 2). The three cases with presumable re-infection after positive baseline serology were all diagnosed in January 2021 after a follow-up (i.e., time from baseline serology to second positive SARS-CoV-2 test) of 198, 200, and 220 days. One of the three HCWs was asymptomatic at time of re-infection. Post hoc measurement revealed positive anti-S antibodies at baseline for all three HCW. For detailed characteristics, see Table 2.

**Table 2:** Characteristics of three participants with positive SARS-CoV-2 nasopharyngeal swab after positive baseline serology

	HCW 1	HCW 2	HCW 3
Sex	Female	Female	Male
Age range at re-infection	50–55	50–55	40–45
Blood group	Unknown	B	B
Comorbidities	None	Pollen allergy	Pollen allergy
Profession	Nurse (assistant)	Nurse (assistant)	Nurse (assistant)
Patient contact	Administrative	Administrative/caring	Administrative
First positive PCR	March 2020	Date unknown	March 2020
Date baseline serology	July 2020	July 2020	June 2020
Anti-spike titers (units/ml), cut-off for positivity 0.8	25.8	244	30
Second positive test (method)	January 2021 (PCR)	January 2021 (PCR)	January 2021 (PCR)
Days baseline to second episode	198	200	220
Days first to second episode	297	Unknown	314
Negative tests between first and second episode	None	None	One
Symptoms second test	Sore throat, coryza, headache, limb/muscle pain, eye irritation, weakness	None	Headache, dizziness, diarrhea, shivering, limb/muscle pain, eye irritation, weakness
Exposure	Patients	Patients	Patients, household
Number or household members	One	One	One
COVID-19 vaccine	None	None	First dose in week of positive test

*Abbreviations*: *HCW* healthcare worker, *PCR* polymerase chain reaction, *COVID-19* coronavirus disease 2019

**Fig. 3:**
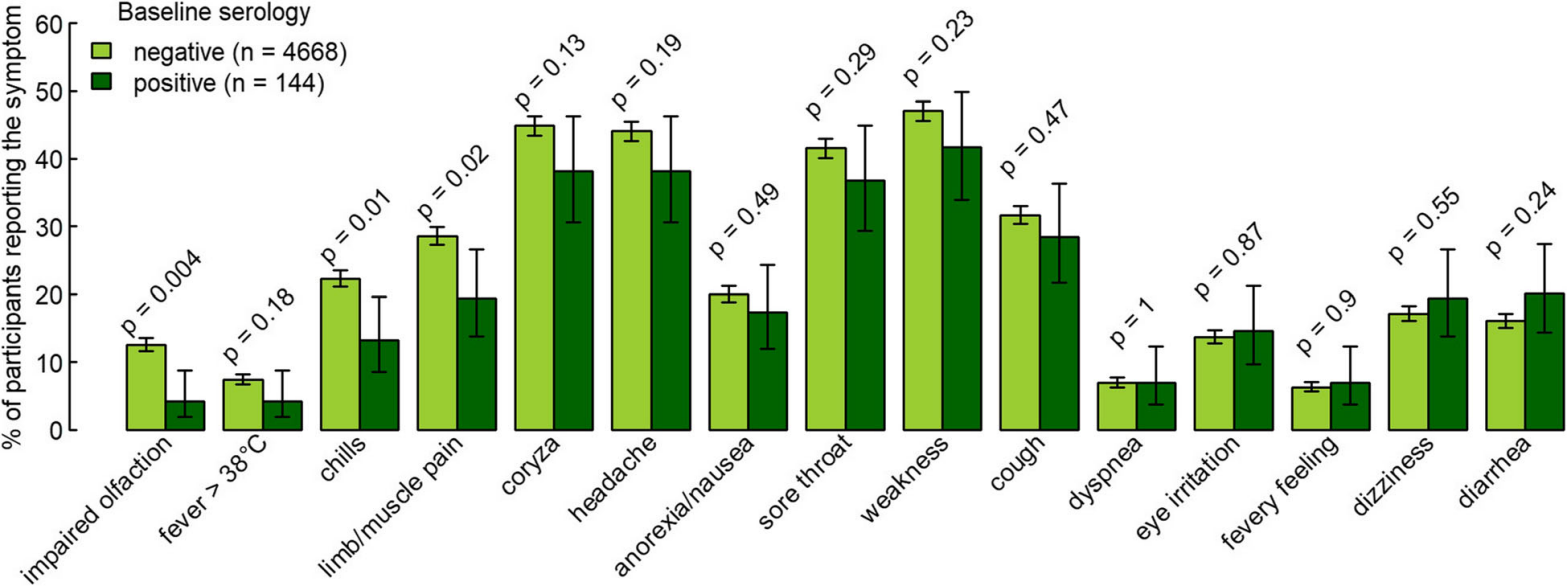
Frequency of symptoms during a median follow-up of 7.9 months based on 101,233 weekly diaries from participants with (*n* = 144) and without (*n* = 4668) specific SARS-CoV-2 antibodies at baseline. Symptoms are sorted by increasing risk ratio. Error bars show 95% Wilson confidence intervals. *P* values calculated by two-sample proportion test

Among 4812 participants, 2879 HCW (59.8%) reported at least one symptom during follow-up, whereas the others (*n* = 1933) remained without symptoms during follow-up. Symptoms were reported by 79/144 (54.9%) of the initially seropositive participants and by 2800/4668 (60%) of the initially seronegative participants (*P* = 0.25) (Fig. 1). The total number of reported symptoms was not significantly different in baseline seropositive compared to seronegative individuals (median of 5 vs. 6 symptoms, *P* = 0.30). Out of 15 different symptoms, ten were reported less frequently by participants who were seropositive at baseline, although the difference was statistically significant only for impaired olfaction/taste (RR 0.33, 95% CI 0.15–0.73, *P* = 0.004), chills (RR 0.59, 95% CI 0.39–0.90, *P* = 0.01), and limb/muscle pain (RR 0.68 95% CI 0.49-0.95, *P* = 0.02) (Fig. 3, Table 3).

**Table 3:** Frequency of individual symptoms (*n* and % of participants who reported it at least once during study participation more than 2 weeks after baseline and prior to vaccination) in relation to baseline serology (total of 4812 participants). Symptoms are sorted by increasing risk ratio (RR)

Symptom	*N* (%) of HCWs reporting a symptom		RR (95% CI)	*P* value prop.test*	*P* value adjusted**
	Seronegatives	Seropositives			
	*N* = 4668	*N* = 144			
Olfaction/taste impaired	588 (12.6%)	6 (4.2%)	0.33 (0.15–0.73)	0.004	0.001
Fever > 38 °C	348 (7.5%)	6 (4.2%)	0.56 (0.25–1.23)	0.185	0.101
Chills	1040 (22.3%)	19 (13.2%)	0.59 (0.39–0.90)	0.013	0.005
Limb/muscle pain	1335 (28.6%)	28 (19.4%)	0.68 (0.49–0.95)	0.021	0.01
Coryza/nasal congestion	2094 (44.9%)	55 (38.2%)	0.85 (0.69–1.05)	0.134	0.083
Headache	2058 (44.1%)	55 (38.2%)	0.87 (0.70–1.07)	0.187	0.126
Anorexia/nausea	935 (20%)	25 (17.4%)	0.87 (0.60–1.24)	0.494	0.393
Sore throat	1940 (41.6%)	53 (36.8%)	0.89 (0.71–1.10)	0.292	0.207
Weakness	2196 (47%)	60 (41.7%)	0.89 (0.73–1.08)	0.235	0.162
Cough	1478 (31.7%)	41 (28.5%)	0.90 (0.69–1.17)	0.471	0.37
Dyspnea	326 (7%)	10 (6.9%)	0.99 (0.54–1.82)	1	0.962
Eye irritation	641 (13.7%)	21 (14.6%)	1.06 (0.71–1.59)	0.866	0.805
Fevery feeling	295 (6.3%)	10 (6.9%)	1.10 (0.60–2.02)	0.897	0.789
Dizziness	801 (17.2%)	28 (19.4%)	1.13 (0.81–1.59)	0.546	0.509
Diarrhea	751 (16.1%)	29 (20.1%)	1.25 (0.90–1.74)	0.236	0.222

*Abbreviations*: *RR* risk ratio, *CI* confidence interval

*Two-sample proportion test

**Based on a logistic regression model including the log-transformed number of questionnaires submitted as covariate

For the analysis of symptom specificity, we included 532 episodes with positive and 1527 with negative test results. Almost all (14 out of 15) symptoms were less frequently reported during episodes with a positive NPS (compared to episodes with negative NPS result). The symptoms which discriminated best between episodes with positive and negative NPS were impaired olfaction/taste (OR 22.2 95% CI 17.1–29.1, *P* < 0.001), limb/muscle pain (OR 4.7 95% CI 3.8–5.9, *P* < 0.001), and weakness (OR 4.4 95% CI 3.1–6.4, *P* < 0.001) (Additional file 2: Figure S2, Additional file 3: Table S1).

For validation of swab results, we cross-checked self-reported NPS results for a subgroup of participants (from the largest participating institution). We found that 150 out of 174 presumable positive NPS were indeed documented in the database of the division of occupational medicine. The remaining HCWs most likely had a positive NPS outside of their working place. On the other hand, none of the randomly selected 175 HCWs reporting only negative NPS results was found to have a positive NPS in the database.

## Discussion

In this prospective cohort of over 4800 HCW followed during the second wave in Switzerland, we demonstrate that the presence of anti-nucleocapsid SARS-CoV-2 antibodies at baseline not only reduced the risk of positive nasopharyngeal SARS-CoV-2 tests, but also the occurrence of COVID-19 specific symptoms such as loss of smell and limb or muscle pain. The follow-up of almost 8 months, the large sample size, the systematic collection of symptoms, and the excellent questionnaire response rate are among the strengths of the study.

These results add to the mounting evidence that specific antibodies protect against subsequent SARS-CoV-2 infection. In the study of Lumley et al., the adjusted incidence rate ratio for seropositive HCW to have a positive PCR (median follow-up 6 months) was 0.11 compared to those without antibodies [4]. Within a cohort of over 3.2 million US patients, Harvey et al. found a ratio of 0.10 of positive PCRs among those with vs. those without positive antibody test at baseline (for tests performed > 90 days after baseline) [8]. Among over 43000 people with a positive antibody test (median follow-up 4 months) from Qatar, the estimated efficacy of natural infection against re-infection was above 90% [9]. Also, data from a population-based study (> 500,000 people) conducted in Denmark suggest that protection after natural SARS-CoV-2 infection was 80% after 6 months (but only 47% for adults aged 65 years or older) [10]. A retrospective propensity-score matched cohort study from Western Switzerland found a 94% reduction in the hazard of having a positive SARS-CoV-2 test for seropositive individuals [11]. In a large HCW cohort from England, a previous history of SARS-CoV-2 infection was associated with a 84% lower risk of infection (median follow-up 7 months) [12]. Likewise, in a university student population from the USA undergoing repeat mandatory testing, an 84% protection from SARS-CoV-2 infection was found [13]. Also, in more than 3000 prospectively followed male US Marine recruits, subsequent infection was found about one fifth compared with seronegative individuals [14]. Although our median follow-up of 8 months is among the longest compared with other studies, our point estimate (RR 0.22) for protection from re-infection with SARS-CoV-2 is perfectly in line with these results. Our findings are further supported by a modeling study of the progression over 250 days of neutralizing antibodies following infection or vaccination and their protective role against symptomatic SARS-CoV-2 infection [15].

Previous studies have mainly looked at the incidence of confirmed SARS-CoV-2 infection to assess the risk of re-infection [8–10]. This approach has the inherent limitation that people who are seropositive might be less likely to undergo NPS testing, as seen in our data. In addition to the collection of NPS test results, we therefore used a second approach to assess the protective effect of seropositivity on subsequent COVID-19. Using weekly symptom frequency from diaries as proxy for COVID-19 allows comparing incidence of COVID-19 irrespective of whether patients undergo NPS testing or not. Of course, this signal is being diluted by infections caused by other respiratory viruses, especially for symptoms like coryza, sore throat, cough, or fever. However, certain symptoms such as loss of taste or smell and myalgias have already been shown by others to be more specific for COVID-19 [16, 17]. The fact that exactly these symptoms occurred less frequently among those with antibodies at baseline supports our finding of a reduced risk for SARS-CoV-2 re-infection in this group. A limitation of this approach is that some participants might suffer from persisting COVID-19 symptoms (i.e., long-COVID). In particular, loss of smell has been shown to persist for several weeks in a certain proportion of COVID-19 patients [18]. If we had excluded seropositive participants who already reported this symptom at baseline, the effect would have even been more pronounced.

Worryingly, re-infections with phylogenetically different SARS-CoV-2 strains are increasingly being reported [19]. A recent report documented severe re-infection with the “new variant” VOC-202021/01 8 months after documented primary infection, even in the presence of anti-nucleocapsid antibodies and in the absence of overt immunosuppression [20]. We also observed three nurse assistants with presumable SARS-CoV-2 re-infection approximately 300 days after first infection and about 6 months after documented seroconversion. Post hoc testing revealed that all three re-infected cases had positive anti-spike titers at baseline. Of note, one of the nurses with highest anti-S did not report any symptoms at time of the test. Although we do not have any sequencing data for these particular viral strains, re-infections occurred in January 2021, when the proportion of the B.1.1.7 (alpha variant) was estimated to account for less than 20% of all SARS-CoV-2 isolates in Switzerland and when B.1.617.2 (delta variant) was not circulating at all [21]. Importantly, recent data suggest that protection from the SARS-CoV-2 delta variant, which is currently the predominant strain in most Western countries, might be clearly below 80% after natural infection [22]. Continuing follow-up of our and other cohorts will reveal the long-term protective effect of specific antibodies against SARS-CoV-2 and its emerging variants. Another open research question regards the long-term protective effect of vaccine-induced immunity, which might be higher compared to natural infection [15, 23].

A limitation of our study is the fact that nasopharyngeal SARS-CoV-2 testing was not routinely performed. Therefore, asymptomatic carriage of SARS-CoV-2 cannot be excluded in those with antibodies at baseline. We also used SARS-CoV-2 anti-nucleocapsid antibody titers and not anti-spike antibodies, which have been shown to correlate better with virus neutralization, to define seropositivity. Post SARS-CoV-2 infection, anti-N antibodies are detected equally [24] or even more frequently [25] than anti-S antibodies. We therefore suggest that our results represent, or somewhat underestimate, the true protective effect mediated by anti-S antibodies although all three re-infected cases had also detectable anti-spike antibodies at baseline. Swab results and symptoms were self-reported. Because validation of some of the swab results showed mostly consistent results, we consider these self-reported data to be highly reliable. Furthermore, we cannot definitely confirm that the three seropositive HCWs with positive SARS-CoV-2 NPS were indeed re-infected with a new strain. However, the long latency between the episodes, new onset of symptoms (two cases), and a negative PCR between episodes (one case) strongly support our hypothesis of re-infection (rather than persistence of viral RNA for more than 6 months). Another shortcoming of this study is that SARS-CoV-2 specific cellular immunity was not evaluated. This is mainly due to the fact that these measurements are still very time-consuming and cost-intensive in a large population. Thus, data are scarce so far. However, specific T cells most likely also contribute decisively to protection against SARS-CoV-2 infection [26]. Immunity mediated by specific T cells can be present even if there have never been signs of disease and antibodies are absent [27, 28]. In consequence, measuring antibodies alone, such as in our study, underestimates protection against COVID-19 in a population. Finally, we acknowledge that the diagnostic sensitivity of SARS-CoV-2 antigen tests is only around 80%; because 36% of NPS were indeed done by antigen test, the effect size observed in our study might be slightly overestimating the true protective effect.

## Conclusion

We conclude that anti-nucleocapsid antibodies acquired after natural infection convey an approximately 80% protection against symptomatic SARS-CoV-2 infection, at least for a period of 8 months and in a setting of unvaccinated HCW where “new variant” mutations were not widely present at the end of follow-up. Syndromic surveillance for specific COVID-19 symptoms allows estimating the probability of SARS-CoV-2 re-infection irrespective of whether participants undergo NPS testing or not.

## Supplementary Information

**Additional file 1: Figure S1.** SARS-CoV-2 epidemiology in Switzerland and timing of the study procedures.

**Additional file 2: Figure S2.** Percentage of participants reporting individual symptoms at the time of either negative or positive nasopharyngeal swabs, with 95% Wilson confidence intervals and with *p*-values from Fisher’s exact tests. Symptoms are sorted by decreasing odds ratio (OR) for occurrence together with a positive swab (see also Additional file 3: Table S1).

**Additional file 3.**

## Data Availability

The dataset used in this publication is available from the corresponding author on reasonable request.

## Abbreviations

AGP: aerosol-generating procedure
BMI: body mass index
CI: Confidence interval
COVID-19: Coronavirus disease 2019
ECLIA: Electro-chemiluminescence immunoassay
HCW: Healthcare worker
HR: Hazard ratio
IQR: Interquartile range
NPS: Nasopharyngeal swab
OR: Odds ratio
PCR: Polymerase chain reaction
RR: Risk ratio
SARS-CoV-2: Severe acute respiratory syndrome
