# Supplementary figures and images for: Impact of baseline SARS-CoV-2 antibody status on syndromic surveillance and the risk of subsequent COVID-19—a prospective multicenter cohort study
Source: BMC Med. 2021 Oct 14;19:270. doi: 10.1186/s12916-021-02144-9 (PMC8514323; doi:10.1186/s12916-021-02144-9)

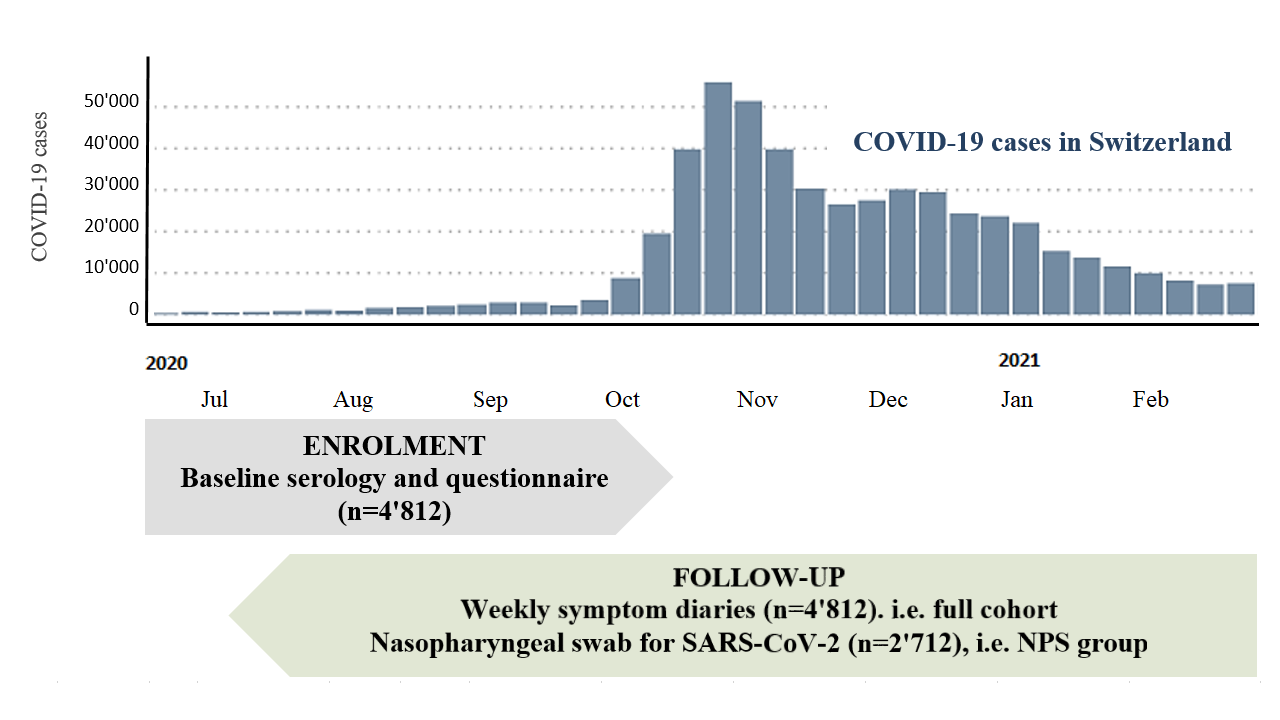

Supplement: Supplementary file 1 — Additional file 1: Figure S1. SARS-CoV-2 epidemiology in Switzerland and timing of the study procedures. [file 12916_2021_2144_MOESM1_ESM.tif]

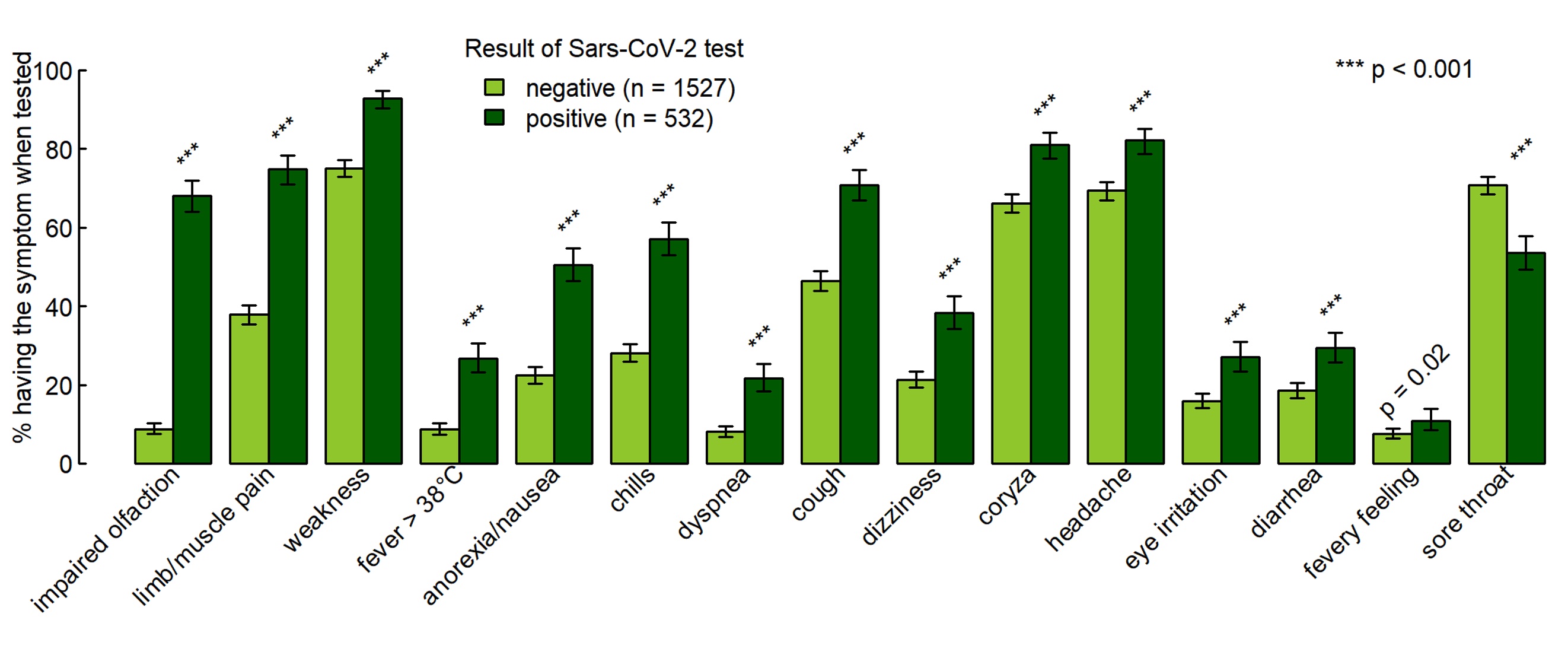

Supplement: Supplementary file 2 — Additional file 2: Figure S2. Percentage of participants reporting individual symptoms at the time of either negative or positive nasopharyngeal swabs, with 95% Wilson confidence intervals and with p-values from Fisher’s exact tests. Symptoms are sorted by decreasing odds ratio (OR) for occurrence together with a positive swab (see also Additional file 3: Table S1). [file 12916_2021_2144_MOESM2_ESM.jpg]
