# Supplementary material for: Impact of baseline SARS-CoV-2 antibody status on syndromic surveillance and the risk of subsequent COVID-19—a prospective multicenter cohort study
Source: BMC Med. 2021 Oct 14;19:270. doi: 10.1186/s12916-021-02144-9 (PMC8514323; doi:10.1186/s12916-021-02144-9)
Supplement: Supplementary file 3 — Additional file 3. [file 12916_2021_2144_MOESM3_ESM.pdf]

Table S1. Frequency of individual symptoms around the time of a nasopharyngeal swab in relation to the test result (negative or positive), considering only participants who reported at least one symptom at this time. Symptoms are sorted by decreasing odds ratio (OR).

Symptom	n (%) of HCWs reporting a symptom		OR (95% CI)*	*p*-value
	negative swab N=1527	positive swab N=532		Fisher
Olfaction/taste impaired	134 (8.8%)	362 (68%)	22.07 (17.03-28.79)	<0.001
Limb/muscle pain	578 (37.9%)	398 (74.8%)	4.87 (3.89-6.13)	<0.001
Weakness	1146 (75%)	494 (92.9%)	4.32 (3.03-6.3)	<0.001
Fever > 38°C	133 (8.7%)	142 (26.7%)	3.81 (2.91-5)	<0.001
Anorexia/nausea	342 (22.4%)	269 (50.6%)	3.54 (2.86-4.39)	<0.001
Chills	429 (28.1%)	304 (57.1%)	3.41 (2.77-4.21)	<0.001
Dyspnea	123 (8.1%)	115 (21.6%)	3.15 (2.36-4.19)	<0.001
Cough	708 (46.4%)	377 (70.9%)	2.81 (2.26-3.5)	<0.001
Dizziness	326 (21.3%)	204 (38.3%)	2.29 (1.84-2.85)	<0.001
Coryza/nasal congestion	1011 (66.2%)	431 (81%)	2.18 (1.7-2.8)	<0.001
Headache	1059 (69.4%)	437 (82.1%)	2.03 (1.58-2.63)	<0.001
Eye irritation	242 (15.8%)	144 (27.1%)	1.97 (1.54-2.51)	<0.001
Diarrhea	283 (18.5%)	156 (29.3%)	1.82 (1.44-2.3)	<0.001
Fevery feeling	115 (7.5%)	58 (10.9%)	1.5 (1.06-2.12)	0.02
Sore throat	1080 (70.7%)	285 (53.6%)	0.48 (0.39-0.59)	<0.001

* estimated through the procedure implemented in Fisher's exact test
